# Leptin promotes rapid dynamic changes in hippocampal dendritic morphology

**DOI:** 10.1016/j.mcn.2007.05.001

**Published:** 2007-08

**Authors:** Dervla O’Malley, Neil MacDonald, Sarah Mizielinska, Christopher N. Connolly, Andrew J. Irving, Jenni Harvey

**Affiliations:** Neurosciences Institute, Division of Pathology and Neuroscience, Ninewells Hospital and Medical School, University of Dundee, Dundee, DD1 9SY, UK

**Keywords:** Leptin, Dendritic filopodia, Synaptic plasticity, MAPK, Hippocampus, Real-time imaging

## Abstract

Recent studies have implicated the hormone leptin in synaptic plasticity associated with neuronal development and learning and memory. Indeed, leptin facilitates hippocampal long-term potentiation and leptin-insensitive rodents display impaired hippocampal synaptic plasticity suggesting a role for endogenous leptin. Structural changes are also thought to underlie activity-dependent synaptic plasticity and this may be regulated by specific growth factors. As leptin is reported to have neurotrophic actions, we have examined the effects of leptin on the morphology and filopodial outgrowth in hippocampal neurons. Here, we demonstrate that leptin rapidly enhances the motility and density of dendritic filopodia and subsequently increases the density of hippocampal synapses. This process is dependent on the synaptic activation of NR2A-containing NMDA receptors and is mediated by the MAPK (ERK) signaling pathway. As dendritic morphogenesis is associated with activity-dependent changes in synaptic strength, the rapid structural remodeling of dendrites by leptin has important implications for its role in regulating hippocampal synaptic plasticity and neuronal development.

## Introduction

It is well documented that leptin regulates energy homeostasis via its actions on specific hypothalamic nuclei (Jacob et al., 1997). Hypothalamic leptin receptors also play an important role in controlling thermogenesis, neuroendocrine function and bone formation (Freidman and Halaas, 1998; Elmquist et al., 1998b; Karsenty, 2001). However, leptin receptors are widely expressed in many brain regions including the hippocampus, cerebellum, brain stem and amygdala (Hakansson et al., 1998; Elmquist et al., 1998a; Shanley et al., 2002), suggesting that leptin plays a role in diverse CNS functions. Recent studies have implicated leptin in associative learning and memory as leptin-insensitive (db/db mice and fa/fa rats) rodents display impairments in hippocampal long-term potentiation (LTP) and long-term depression (LTD), as well as deficits in spatial memory tasks (Li et al., 2002). Direct administration of leptin into the hippocampus enhances LTP in vivo *(*Wayner et al., 2004). At the cellular level, leptin converts hippocampal short lasting potentiation (STP) into LTP; an action likely to reflect enhanced NMDA receptor function (Shanley et al., 2001). Leptin also contributes to synaptic plasticity changes in the hypothalamus as the efficacy of inhibitory and excitatory synaptic transmission is altered in leptin-deficient ob/ob mice (Pinto et al., 2004). During neuronal development, leptin also plays a pivotal role: leptin receptors are expressed at high levels in neonatal rodent brains (Morash et al., 2001), and reductions in brain weight and protein content are evident in leptin-deficient or -insensitive rodents (Ahima et al., 1999). More recent studies indicate that leptin participates in development of the hypothalamus as specific projection pathways are disrupted in leptin-deficient ob/ob mice (Bouret et al., 2004).

Several lines of evidence indicate that structural, as well as biochemical changes underlie activity-dependent synaptic plasticity in the brain. Indeed changes in the morphology and/or density of dendritic spines contribute to enhanced synaptic efficacy following hippocampal LTP (Yuste and Bonhoeffer, 2001). Moreover, neurotrophic factors, like brain-derived neurotrophic factor (BDNF), further refine synaptic connections during activity-dependent synaptic plasticity (Tyler et al., 2002; Schinder and Poo, 2000). Indeed, BDNF, via a MAPK-dependent process, promotes changes in the morphology and density of dendritic spines in CA1 pyramidal neurons (Tyler and Pozzo-Miller, 2003), which may contribute to this refining process. As leptin has neurotrophic actions in the hypothalamus (Bouret et al., 2004), it is possible that changes in dendritic morphology may also contribute to the changes in hippocampal synaptic efficacy induced by leptin. In this study, we present the first compelling evidence that leptin rapidly increases the density and motility of dendritic filopodia in hippocampal neurons. Moreover, this effect is associated with the formation of new synaptic connections as leptin rapidly increased the number of hippocampal synapses. These findings have important implications for the role of this hormone in hippocampal synaptic plasticity and neuronal development.

## Results

### Leptin increases the number of dendritic filopodia in hippocampal neurons

It is well documented that dendritic filopodia are highly motile structures and the motility and/or number of filopodia can be influenced by a range of extrinsic factors, such as neurotrophins (Munno and Syed, 2003). In order to examine if leptin alters the density of filopodia, we compared the number of filopodia extending from dendrites of cultured hippocampal neurons (6–12 DIC) under control conditions or following exposure to leptin (50 nM). Filopodia were identified in fixed, permeabilized cells using Alexa 488-phalloidin labeling. Dendritic processes were identified by their morphology and MAP2 staining. In the absence of leptin, the density of filopodia was generally low, with on average between 2 and 9 filopodia (mean 2.58 ± 0.14; *n* = 129) detected on 50 μm segments of randomly selected proximal dendritic processes. However, in neurons treated with leptin (50 nM for 30 min) the filopodial density markedly increased (309 ± 34% of control; mean density of 8.00 ± 0.45; *n* = 23; *P* < 0.001), suggesting that leptin promotes outgrowth or stabilization of filopodia from dendritic processes. In contrast, exposure to the inactive boiled leptin peptide (30 min) had no effect on the number of dendritic filopodia such that the mean density of filopodia in control conditions and following exposure to the inactive peptide were 3.19 ± 0.21 and 3.04 ± 0.16, respectively (*n* = 29; *P* > 0.05). In order to demonstrate that the changes in dendritic morphology induced by leptin were attributable to activation of leptin receptors, the effects of leptin were also assessed in neurons transfected with leptin receptor siRNA to reduce leptin receptor expression. In order to aid visual identification of siRNA-targeted cells, neurons were also co-transfected with EGFP cDNA. Under control conditions (normal leptin receptor expression), application of leptin (50 nM; 30 min) resulted in a marked increase in the density of dendritic filopodia (mean density of 4.97 ± 0.25; *n* = 29; *P* < 0.05) relative to control (mean density of 1.58 ± 0.18; *n* = 32). In contrast, in neurons with reduced leptin receptor expression, the ability of leptin (50 nM; 30 min) to alter the density of dendritic filopodia was significantly reduced such that the mean density of filopodia was 1.37 ± 0.15 (*n* = 36) and 1.48 ± 0.13 (*n* = 36; *P* > 0.05) in the absence and presence of leptin, respectively. These data suggest that the alterations in dendritic morphology induced by leptin are mediated by activation of leptin receptors as genetic knockdown of leptin receptor expression in hippocampal neurons markedly attenuated the effects of this hormone.

**Fig. 1:**
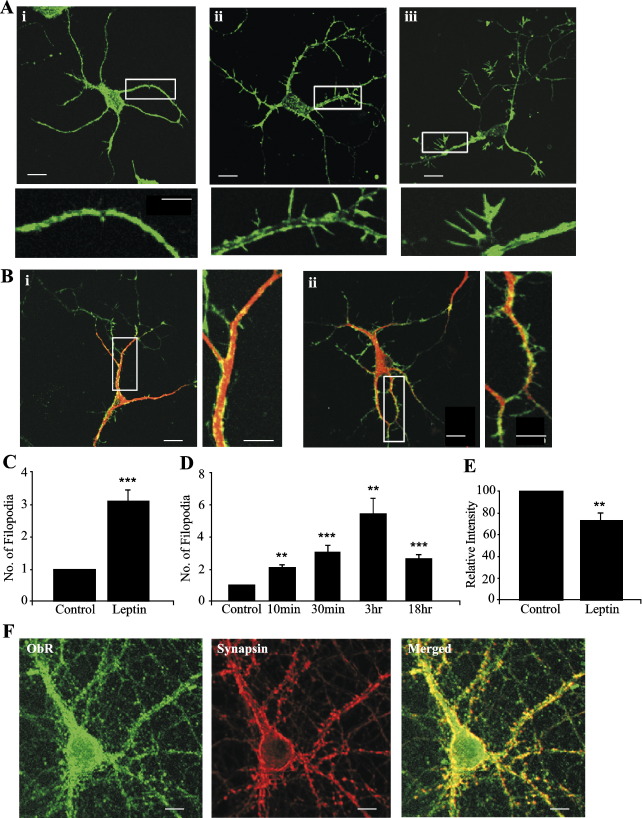
Leptin increases the density of dendritic filopodia in hippocampal neurons. (A) Confocal images of actin staining in hippocampal neurons (6–8 DIC) labeled with Alexa 488-conjugated phalloidin. (Ai) Control neurons display relatively few actin-rich protrusions or growth cones. Exposure of neurons to leptin (50 nM) for 30 min stimulated an increase in the number of filopodia (ii) and growth cones (iii) extending from processes (indicated by arrows). (B) Confocal images of hippocampal neurons (9DIC) dual labeled with Alexa 488-conjugated phalloidin (green) and the somatodendritic marker, MAP2 (red). Leptin (50 nM; 30 min) increased the number of filopodia protruding from dendritic (MAP2-positive) processes (ii) compared to control (i). (C) Histogram illustrating the pooled data of the mean number of dendritic filopodia in control and leptin treated neurons. Leptin stimulates circa a 3-fold increase in the density of filopodia. (D) Histogram illustrating the pooled data of the mean number of dendritic filopodia after 10 min, 20 min, 30 min, 3 h and 18 h exposures to leptin (50 nM). The leptin-induced increase in filopodial density is apparent after only 10 min exposure to leptin and reaches a peak after 3 h. (F) Representative confocal images (i–iii) of leptin receptor (ObR; i) and synapsin-1 (ii) immunoreactivity in 9-day-old hippocampal cultures. The merged image (iii) shows that leptin receptor labeling is highly localized to synapses.

In order to determine the temporal profile of these leptin-dependent events, we compared the effect of leptin (50 nM) over a range of time courses (10 min to 18 h). The effect of leptin (50 nM) occurred rapidly as a significant increase in filopodial density (to 206 ± 14% of control; mean density of 5.45 ± 0.36; *n* = 10; *P* < 0.01) was evident after 10 min incubation with leptin, with a peak increase (to 544 ± 95% of control; mean density of 13.8 ± 2.3; *n* = 12; *P* < 0.01) detected after 3 h exposure (Fig. 1D). Furthermore, increases in the density of filopodia (267 ± 19% of control; mean density of 6.89 ± 0.42; *n* = 12; *P* < 0.001) were observed after 18 h exposure to leptin, suggesting that this action of leptin does not readily desensitize (Fig. 1D). However, the morphology of the filopodia was altered at this time point, being lengthened, with actin-rich growth cones apparent at their leading edges (not illustrated). This suggests that the effects of leptin on filopodial density lead to the formation of new processes.

### Leptin induces actin reorganization in hippocampal neurons

It is well established that the actin cytoskeleton plays a pivotal role in the morphological changes that occur in dendritic filopodia and spines during development and synaptic plasticity (Matus, 2000; Smart and Halpain, 2000). In addition, we have shown that leptin has the ability to promote reorganization of actin filaments in hippocampal neurons (O’Malley et al., 2005). Thus, in order to determine if the leptin-induced formation of new filopodia was accompanied by changes in the actin cytoskeletal architecture, the intensity of Alexa-phalloidin staining in proximal dendrites was examined. In control hippocampal neurons (no leptin), Alexa-phalloidin staining was associated with the plasma membrane and actin rich structures such as filopodia and synapses. Exposure to leptin (50 nM; 30 min) induced a marked reorganization of the actin cytoskeleton such that the intensity of Alexa-phalloidin staining in proximal dendrites was significantly reduced (to 67 ± 8.4% of control; *n* = 25; *P* < 0.05), and this effect of leptin was accompanied by the appearance of actin-enriched dendritic filopodia (Fig. 1E). Thus, these data suggest that leptin promotes redistribution of actin from the dendritic shaft to dendritic filopodia.

### Leptin enhances actin-based motility of filopodial extensions

**Fig. 2:**
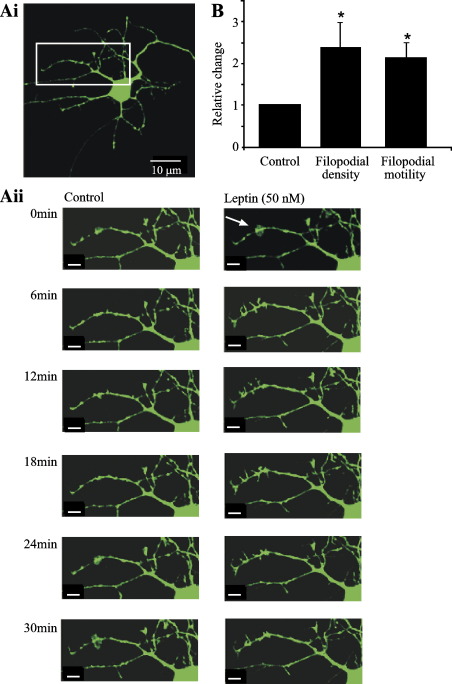
Leptin rapidly increases the number and motility of dendritic filopodia. (A) Confocal images of a hippocampal neuron (11 DIC) transiently transfected with cytosolic EGFP. The region of interest was magnified and the images obtained at various time points (3–30 min) are shown in panel Aii. Under control conditions there are a few filopodia extending from dendrites and these had limited motility. Leptin (50 nM) treatment stimulated a rapid increase in number and motility of filopodia extending from the highlighted dendrite (arrow). (B) Histogram of the pooled data illustrating the relative change in the density and motility of filopodia following exposure to leptin (50 nM). *P < 0.05.

**Fig. 3:**
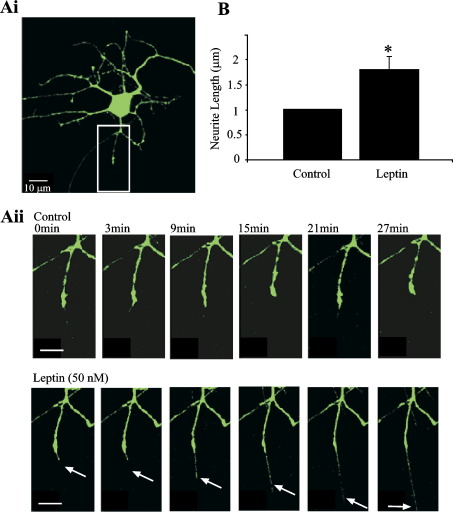
Leptin stimulates an increase in neurite length. (A) Confocal images of a hippocampal neuron (11 DIC) transiently transfected with cytosolic EGFP. The region of interest in panel Ai is magnified and displayed in panel Aii. In control conditions (in the absence of leptin), there is little or no change in the length of the neurite. However, application of leptin (50 nM) to the same neuron rapidly stimulated elongation of the same process. (B) Pooled data illustrating the mean increase in the relative length of protruding neurites following leptin treatment. *P < 0.05.

In order to visualize actin-based structures in living neurones, we transfected cultured hippocampal neurons (up to 12 DIC) with a cytosolic EGFP construct using the cationic lipid, Lipofectamine 2000. In transfected neurons, filopodia were associated with dendrites arising from principle neurons in the culture (pyramidal-shaped, with 2–3 main dendritic branches), and the average number of filopodia (extending from 50 μm proximal regions of randomly selected dendrites) was 7.14 ± 0.58 (*n* = 34 dendrites), whereas the average length of a filopodium was 5.2 ± 0.9 μm (*n* = 69 filopodia). Under control conditions, dendritic filopodia were motile, typically displaying elongation and/or retraction as well as lateral motility. The degree of motility was variable amongst filopodia both within and between different cultures. Thus, in order to quantify motility, the degree of change in the length of individual filopodium was monitored, as described previously (Ziv and Smith, 1996; Tashiro et al., 2003; see Experimental procedures). In control conditions (10–12 DIC; at room temperature), the motility of filopodia (the change in length of a given filopodium over time) was 0.85 ± 0.12 μm/min (*n* = 16). Application of leptin (50 nM) resulted in a rapid (within 3–6 min) increase in both the number of filopodia extending from processes (mean density of 17.9 ±4.4; *n* = 16; *P* < 0.05) and the motility of these extensions (mean motility of 1.9 ± 0.26 μm/min; *n* = 16; *P* < 0.05; Fig. 2). Furthermore, exposure to leptin stimulated an increase in the mean length of extending filopodia-like neurites to 9.7 ± 1.3 μm (*n* = 16; *P* < 0.05; Fig. 3).

### Leptin-induced dendritic morphogenesis requires excitatory synaptic transmission

**Fig. 4:**
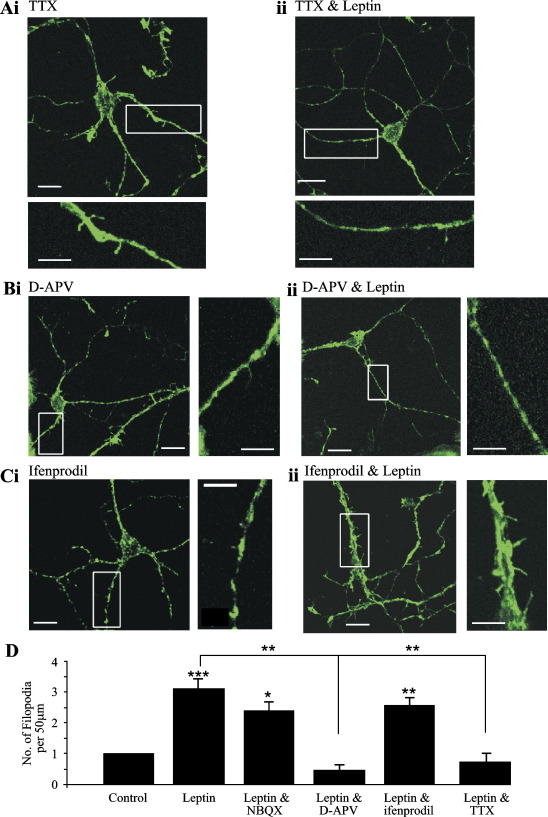
Synaptic activation of NMDA receptors is required for leptin-induced neurite growth. (A–C) Confocal images of hippocampal neurons (12 DIC) labeled with Alexa 488-conjugated phalloidin. The zoomed regions of interest (white box) are depicted either below (A) or alongside (B, C) the parent image. (A) Prior exposure of neurons to TTX (500 nM; 30 min) attenuated the leptin-induced increase in filopodial density (Aii) compared to control (Ai). (Bi) Incubation with the NMDA receptor antagonist, D-APV (50 μM; 30 min), did not alter the filopodial density *per se*. However, prior exposure to D-APV attenuated the leptin-induced increase in the density of dendritic filopodia (Bii). Treatment with the NR2B-selective NMDA receptor antagonist, ifenprodil (10 μM; 30 min), had no effect on the number of dendritic filopodia per se (Ci) nor did it affect the ability of leptin to increase the density of filopodia (Cii). (D) Histogram illustrating the pooled data of the mean number of filopodia in control conditions and in neurons treated with leptin (50 nM; 30 min) alone and in the combined presence of NBQX (2 μM; 30 min), D-APV (50 μM; 30 min), ifenprodil (10 μM; 30 min) and TTX (500 nM; 30 min). *, ** and *** denote *P* < 0.05, *P* < 0.01 and *P* < 0.001, respectively.

In order to assess whether leptin-induced morphogenesis required ongoing synaptic activity we compared the effects of leptin in cultures where action potential-driven synaptic transmission was blocked with tetrodotoxin (TTX; 0.5 μM; 30 min). Under these conditions, the ability of leptin to increase filopodial density was significantly attenuated (mean density of 3.21 ± 0.6 in the presence of TTX and leptin; *n* = 9; *P* > 0.05), indicating that synaptic activity is critical for the morphological changes induced by leptin. It is well documented that excitatory synaptic transmission plays a key role in the outgrowth of neurites. Indeed, dendritic protrusions are stimulated by the synaptic activation of NMDA receptors (Maletic-Savatic et al., 1999) and glutamate modulates the formation and motility of dendritic filopodia (Fischer et al., 2000; McKinney et al., 1999). In this study, NMDA receptor-dependent synaptic activity was also required for the effects of leptin, as NMDA (50 μM D-AP5; *n* = 9), but not AMPA (2 μM NBQX; *n* = 9), receptor blockade prevented the effects of leptin (Figs. 4B, D). Thus, incubation of neurons with NBQX (2 μM; 30 min) had no effect on the density of filopodia per se (mean density of 2.39 ± 0.51; *n* = 9; *P* > 0.05), and it did not significantly attenuate the leptin-induced increase in the number of filopodia (mean density of 5.69 ± 0.76; *n* = 9; *P* < 0.05; Fig. 4D). Application of D-APV (50 μM; 1 h) also had no effect on the mean number of filopodia (mean density of 2.76 ± 0.69; *n* = 10; *P* > 0.05). However, prior incubation with D-APV (50 μM; 30 min) reversed the actions of leptin, such that in the presence of D-APV, leptin (50 nM; 30 min) significantly reduced the mean number of filopodia extending from dendritic processes to 1.35 ± 0.41 (*n* = 9; *P* < 0.05; Figs. 4B, D). This suggests that leptin has diverse actions on dendritic morphogenesis evoking both NMDA receptor-dependent enhancement and NMDA receptor-independent retraction of dendritic filopodia.

**Fig. 5:**
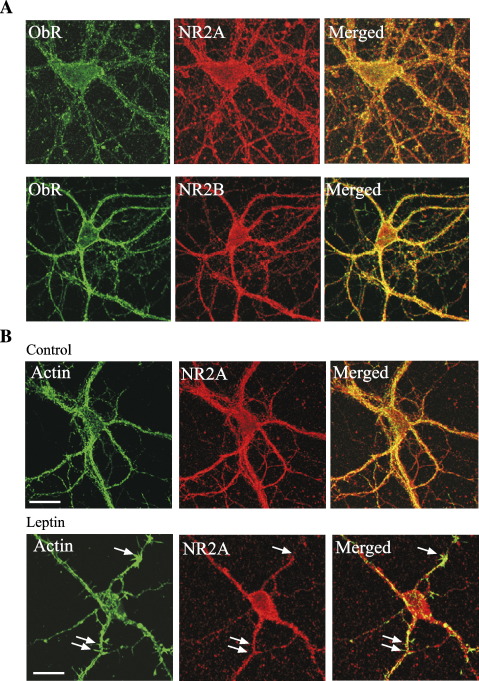
Leptin does not promote the translocation of NR2A subunits into dendritic filopodia. (A) Expression of NR2A and NR2B NMDA receptor subunits in hippocampal cultures. Confocal images of ObR immunolabeling, together with either NR2A (i; 9 DIC) or NR2B (ii; 7DIC) labeling in hippocampal neurons. High levels of NR2A and NR2B are expressed in hippocampal neurons at this stage of development. (B) Confocal images of Alexa phalloidin staining (green), NR2A immunolabeling (red) and the merged images in hippocampal neurons (8 DIC) in control conditions and following exposure to leptin (50 nM; 30 min). Leptin induced an increase in the density of dendritic filopodia; however, this was not associated with any change in the distribution of NR2A staining.

Several lines of evidence indicate that different NMDA receptor NR2 subunits are differentially localized in the hippocampus, such that NR2A-containing NMDA receptors are expressed at synaptic loci, whereas NR2B subunits are predominantly found extrasynaptically (Barria and Malinow, 2002; Tovar and Westbrook, 1999). Recent studies also suggest that activation of NR2A-containing NMDA receptors is a pre-requisite for the induction of hippocampal and cortical LTP, whereas receptors comprising NR2B subunits are activated during LTD (Liu et al., 2004; Massey et al., 2004). Thus, in order to verify that these NMDA receptor subunits are expressed in our hippocampal cultures, the distribution of NR2A and NR2B immunoreactivity was assessed using specific antibodies directed against these subunits. As NMDA receptors are a target for leptin in the hippocampus (Shanley et al., 2001), we also compared the cellular distribution of ObR using dual labeling approaches. In agreement with our previous studies (Shanley et al., 2002), leptin receptor immunoreactivity was detected on cultured hippocampal neurons at all ages examined (7–9 DIC; Figs. 1F and 5A, B). Indeed, high levels of NR2A as well as NR2B immunoreactivity were observed on somata and on processes (Figs. 5A, B) of hippocampal cultures also expressing leptin receptors. In order to verify if activation of putative synaptic (i.e., NR2A-containing) NMDA receptors underlie the neurotrophic actions of leptin, NR2B-containing NMDA receptors were blocked with ifenprodil (Williams, 1993). In contrast to the actions of D-AP5, treatment with ifenprodil (10 μM; 30 min) failed to prevent leptin-induced morphogenesis, as leptin increased the density of filopodia to 6.63 ± 0.57 (*n* = 9; *P* < 0.01; Figs. 4C, D) in the presence of ifenprodil. Thus, it is likely that the activation of putative synaptic, as oppose to extrasynaptic, NMDA receptors is critical for leptin-induced dendritic filopodial outgrowth.

There is growing evidence that trafficking of glutamate receptor plays a critical role in hippocampal synaptic plasticity (Collingridge et al., 2004). Moreover, our previous studies have indicated that leptin has the capacity to increase the cell-surface expression of NMDA receptors (Harvey et al., 2005). Thus, as the leptin-induced formation of dendritic filopodia is dependent on the activation of NR2A-containing NMDA receptors, the effects of leptin on the localization of NR2A subunits was also examined. Under control conditions, NR2A immunolabeling was widely expressed at somatodendritic regions but was rarely expressed in dendritic filopodia (Fig. 5C). Moreover, following exposure to leptin for 30 min, there was no change in the distribution of NR2A subunits, such that the density of NR2A subunits expressed on filopodia was not altered (*n* = 6). These data suggests that leptin does not promote the translocation of NR2A-containing NMDA receptors into dendritic filopodia.

### A MAPK-dependent process underlies leptin-induced increase in dendritic filopodia

**Fig. 6:**
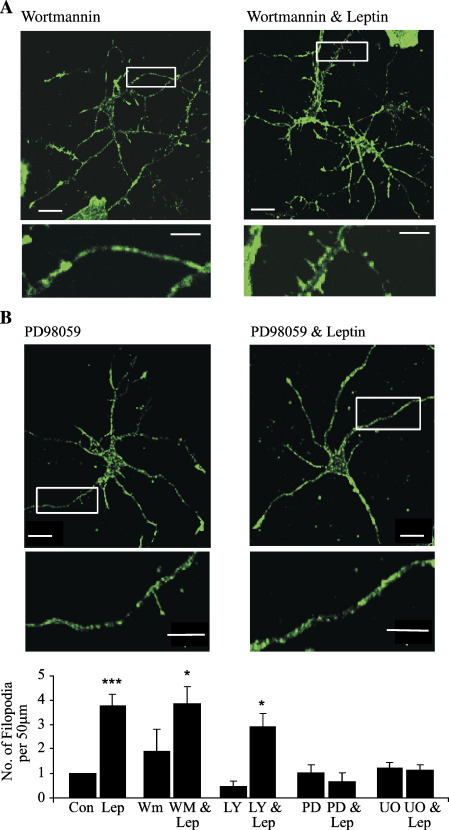
Leptin increases the density of filopodia via a MAPK-, but not PI 3-kinase-, driven pathway. (A, B) Confocal images of F-actin staining in hippocampal neurons (10–11 DIC) labeled with Alexa 488-conjugated phalloidin. The magnified regions of interest (white boxes) are illustrated below the parent images. (Ai) Incubation with wortmannin (50 nM; 1 h) had no effect on the density of filopodia *per se*, and it did not affect the ability of leptin to increase the number of filopodia. In contrast, prior incubation with PD98059 (10 μM; 1 h) attenuated leptin-induced increase in filopodial density (Bii). The density of filopodia was not altered in neurons exposed to PD98059 (Bi). (C) Histogram of pooled data illustrating the mean number of filopodia in control conditions and in neurons exposed to leptin (50 nM; 30 min) both alone and in the presence of wortmannin (WM; 50 nM), LY294002 (LY; 10 μM), PD98059 (PD; 10 μM) and U0126 (UO; 1 μM).

Leptin receptors are class I cytokine receptors (Tartaglia et al., 1995) associated with the activation of PI 3-kinase and MAPK signaling cascades in neurons (Harvey, 2003; Irving et al., 2006). Indeed, the ability of leptin to facilitate NMDA receptor function in the hippocampus involves activation of PI 3-kinase and MAPK-dependent processes (Shanley et al., 2001). Thus, the role of these signaling pathways in leptin-induced morphogenesis was evaluated. Application of the PI 3-kinase inhibitors, LY294002 (10 μM; 1 h) or wortmannin (50 nM; 1 h) had no effect on the density of filopodia per se (LY294002; mean density of 2.45 ± 0.53; *n* = 9; *P* > 0.05 and wortmannin; mean density of 2.66 ± 0.81; *n* = 9; *P* > 0.05). Furthermore, prior incubation with either agent did not attenuate the effects of leptin, as leptin stimulated an increase in the number of filopodia in the presence of either wortmannin (to 9.81 ±0.85; *n* = 9; *P* < 0.05) or LY294002 (to 7.56 ± 0.64; *n* = 9; *P* < 0.05), respectively (Figs. 6A, C). Thus, the leptin-induced morphogenesis is likely to involve a PI 3-kinase-independent process. In contrast, treatment with PD98059 (10 μM for 30 min) to specifically inhibit MEK attenuated the leptin-induced dendritic morphogenesis, such that the mean density of filopodia was 2.29 ± 0.57 (*n* = 10; *P* > 0.05; Figs. 5B, C) following exposure to leptin (50 nM; 30 min). Application of PD98059 alone had no effect on filopodial density per se (mean density of 2.35 ± 0.46; *n* = 9; *P* > 0.05). Similarly, exposure to another MEK inhibitor, U0126 (1 μM), had no effect on the density of filopodia per se (mean density of 2.47 ± 0.56; *n* = 10; *P* > 0.05), but it prevented the leptin-induced morphogenesis, such that the mean density of filopodia following exposure to leptin was 2.51 ± 0.62 (*n* = 11; *P* > 0.05; Fig. 6C) in neurons incubated with U0126 (1 μM; 30 min). Together, these data indicate that the increased density of dendritic filopodia induced by leptin is likely to be driven by activation of a MAPK (ERK)-dependent pathway.

### Leptin promotes formation of functional hippocampal synapses

**Fig. 7:**
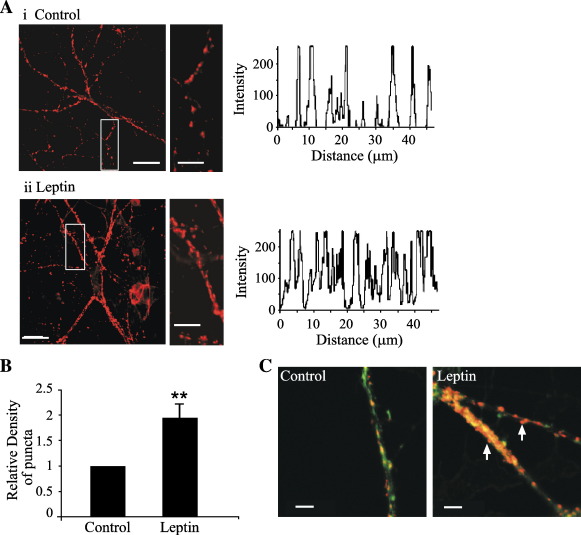
Leptin increases the density of hippocampal synapses. (A) Confocal images of hippocampal neurons (7 DIC) labeled with an anti-synapsin 1 antibody. The magnified regions of interest (white box) and corresponding intensity profile are illustrated to the right of the parent images. Incubation with leptin (50 nM; 30 min) stimulated an increase in the number and intensity of synapsin-1 puncta (Aii) compared to control (Ai). (B) Histogram of the pooled data of the relative intensity of staining in neuronal processes in control conditions and following application of leptin (50 nM; 30 min). (C) Confocal images of hippocampal neurons (14 DIC) dual labeled with Alexa 488-conjugated phalloidin (green) and an anti-synapsin 1 antibody (red). Under control conditions, the neurons display tightly intertwined processes with synapsin 1 labeling. Application of leptin (50 nM; 30 min) to these neurons increased the number and intensity of synapsin 1 puncta as well as the density of spines (arrows).

As the protrusion and motility of dendritic filopodia is thought to be one of the initial stages in the process of synaptogenesis (Munno and Syed, 2003; Fiala et al., 1998), and dendritic filopodia may play an active role in initiating synaptic contacts (Ziv and Smith, 1996), we examined whether leptin-induced neurite outgrowth is correlated with an increase in the number of synapses. Thus, the relative number of presynaptic terminals was assayed by immunostaining of the presynaptic protein, synapsin-1 (Figs. 7A, B). In control neurons (6–7 DIC), synapsin-1 staining was distributed in a punctate manner on both somata and neurites. At this age in culture, application of leptin (50 nM; 30 min) increased the number (to 211 ± 38% of control; *n* = 13; *P* < 0.01) of synapsin-1 puncta (Figs. 7A, B). In older neurons (14 DIC), fewer filopodia extending from dendritic processes were observed and synapsin-1 staining was generally found on the heads of spine-like structures (Fig. 7C), consistent with the appearance of dendritic spines as neuronal networks mature (Fiala et al., 1998). Following leptin (50 nM: 30 min) exposure, both the number of actin-rich spines and the number of synapsin-1-positive puncta increased (to 194 ± 29% of control; *n* = 26; *P* < 0.05; Fig. 7C) in these neurons. Thus, these data suggest that the density of synapses is increased by leptin.

## Discussion

Evidence is accumulating that morphological changes occur during activity-dependent synaptic plasticity that are likely to contribute to alterations in synaptic strength (Maletic-Savatic et al., 1999; Toni et al., 1999; Yuste and Bonhoeffer, 2001). Previous studies have implicated the hormone leptin in hippocampal synaptic plasticity (Shanley et al., 2001; Li et al., 2002; Wayner et al., 2004). However, it is unclear if leptin has the ability to induce morphological changes. Here we show that in hippocampal neurons leptin increased both the motility and density of dendritic filopodia; an effect that occurred rapidly being evident within 10 min exposure to leptin. During prolonged exposure to leptin this process resulted in filopodial stabilization and is likely to result in the formation of new neurites and synaptic contacts. Indeed, the alterations in the number and motility of dendritic filopodia induced by leptin were associated with the formation of new synaptic connections as increases in the labeling of presynaptic components of synapses was evident circa 15–20 min after dendritic morphogenesis.

Numerous studies have reported that synaptic activity, in particular glutamatergic synaptic transmission, plays a key role in both the initiation and stabilization of dendritic filopodia (Munno and Syed, 2003; McKinney et al., 1999). Similarly, ongoing synaptic transmission was required for leptin-induced morphogenesis as blockade of action potential-driven synaptic transmission with TTX prevented the actions of leptin. Moreover, NMDA receptor-dependent activity, in particular the synaptic activation of NR2A-containing receptors, was critical for leptin-induced morphogenesis as the competitive NMDA receptor antagonist, D-AP5, but not the NR2B-selective inhibitor ifenprodil, attenuated the actions of leptin.

It is well established that PI 3-kinase-and MAPK-driven signaling cascades are rapidly activated downstream of neuronal leptin receptors (Harvey, 2003; Niswender et al., 2001; Irving et al., 2006). Indeed in the hippocampus, facilitation of NMDA receptor-dependent function by leptin is mediated by both PI 3-kinase and MAPK (Shanley et al., 2001). In contrast, in this study the effects of leptin on dendritic morphology were dependent solely on the MAPK signaling cascade, as inhibitors of MEK completely attenuated the effects of leptin on filopodial density. Although it is likely that leptin promotes activation of MAPK via stimulation of the ubiquitous Ras-Raf pathway (Blenis, 1993), the possibility that a distinct upstream pathway couples leptin receptor activation to MEK/MAPK stimulation cannot be excluded as leptin is able to trigger the MAPK cascade via either tyrosine phosphorylation of the JAK2-receptor complex or independently of receptor phosphorylation (Bjorbaek et al., 2001). As the downstream signaling in both pathways requires an intact catalytic domain of SHP-2, one possibility is that ERK activation by leptin receptors involves the SHPS1-SHP-2 complex that is known to be activated by integrin (Fujioka et al., 1996). It is well documented that STAT3-dependent signaling, which in turn promotes changes in gene transcription, plays an important role in a number of leptin driven hypothalamic functions including body weight regulation, lactation and regulation of thyroid function (Bates and Myers, 2004). Thus, although a role for STAT3 signaling cannot be completely excluded in this study, due to the rapid nature of the morphological changes induced by leptin, it is unlikely that STAT-dependent gene transcriptional changes play a significant role in this process.

Recent studies have implicated a number of neurotrophins and growth factors in dendritic remodeling, which may in turn underlie their ability to modulate synaptic plasticity (Ethell and Pasquale, 2005). For instance, exogenous application of BDNF evokes similar morphological changes in dendrites as those reported to occur following induction of hippocampal LTP including increased spine density and the appearance of new spines McAllister et al, 1995). Similarly, insulin-like growth factor-1 (IGF-1), which signals via similar signaling pathways to leptin, promotes dendritic morphogenesis in cortical neurons (Niblock et al., 2000). However, in contrast to the present study, the effects of these neurotrophic factors on dendritic morphology occur on a slower time scale than those of leptin. Indeed, remodeling of cortical dendrites is evident after 24–36 h exposure to BDNF or IGF-1 (McAllister et al, 1995; Niblock et al., 2000), whereas in the present study the morphological changes induced by leptin occurred within 10 min. It is perhaps interesting to note that a recent study has shown that integrins, a component of the extracellular matrix, also have the ability to rapidly remodel hippocampal dendrites (Shi and Ethell, 2006) as well as playing an important role in synaptic plasticity (Chun et al., 2001). Moreover, in a manner similar to the effects of leptin in this study, the structural dendritic changes induced by integrins occur rapidly (within 30 min), involve reorganization of actin filaments and require NMDA receptor activation (Shi and Ethell, 2006).

There is evidence that leptin alters neuronal morphology in other brain regions. Indeed, leptin promotes neurite outgrowth from a specific population of neurons in the arcuate nucleus of the hypothalamus (Bouret et al., 2004). However, the trophic actions of leptin on hypothalamic neurons were not evident until at least 72 h exposure to this hormone (Bouret et al., 2004) and are likely to reflect a process subsequent to the more rapid effects of leptin on filopodial motility and synaptic density reported in the present study. Similarly the ability of leptin to increase axonal growth cone size in cortical neurons occurred on a slower time scale as this effect was only apparent after 72 h exposure to this hormone (Valerio et al., 2006).

In this study, the dendritic morphogenesis induced by leptin was paralleled by a significant reduction in the intensity of Alexa-phalloidin staining in dendrites suggesting that this process involves dynamic alterations in the actin cytoskeletal architecture. This is perhaps not surprising as it is well documented that dendritic filopodia and spines are actin-rich structures and that formation and loss of these processes as well as their morphological plasticity involve reorganization of the actin cytoskeleton (Halpain et al., 1998; Fischer et al., 1998). Moreover, several lines of evidence indicate that alterations in actin dynamics also underlie hippocampal activity-dependent synaptic plasticity (Dillon and Goda, 2005). It is interesting to note that the ability of leptin to modulate hippocampal large-conductance Ca^2+^-activated K^+^(BK) channels is also dependent on actin filament reorganization (O’Malley et al, 2005). However, in contrast to the present study, a PI 3-kinase driven process mediates actin filament disruption and BK channel activation by leptin (O’Malley et al, 2005; Shanley et al., 2002). As BK channel activation by leptin involves a membrane-delimited process (Shanley et al., 2002), and leptin driven PI 3-kinase activation results in a localized, membrane-associated increase in phosphatidylinositol-3,4,5-triphosphate (PtdIns(3,4,5)P_3;_ O’Malley et al, 2005), leptin signaling via this route is likely to result in a highly compartmentalized reorganization of actin filaments. In contrast, as a number of downstream targets for MAPK are present throughout dendritic and somatic regions of hippocampal neurons (Thomas and Huganir, 2004), it is likely that MAPK activation by leptin results in more global changes in actin dynamics. Thus, this raises the interesting possibility that by utilizing distinct signaling pathways leptin evokes spatially segregated alterations in actin dynamics, which in turn may differentially influence not only its cellular target but also its subsequent impact on neuronal function.

The structural changes induced by leptin in this study show many parallels to activity-dependent long-term morphological changes in dendrites, such as those observed following hippocampal LTP. Indeed, leptin-induced an increase in the number of actin-rich spines, which parallels the morphological change that accompany hippocampal LTP (Fukazawa et al., 2003). In a manner similar to LTP, an increase in the density of filopodia was evident after exposure to leptin. The time course of LTP-induced alterations in dendritic morphology was similar to that evoked by leptin, as dendritic morphogenesis can occur around 10 min after tetanic stimulation (Maletic-Savatic et al., 1999). As with leptin in this study, high frequency stimulation paradigms that promote rapid growth of dendritic filopodia require the synaptic activation of NMDA receptors (Maletic-Savatic et al., 1999), and NMDA receptor-dependent forms of hippocampal LTP are associated with a rapid increase in the number of synapses (Toni et al., 1999; Antonova et al., 2001). Several lines of evidence indicate that MAPK activation is also essential for the structural changes that occur during activity-dependent synaptic plasticity. Indeed, sustained activation of MAPK by repetitive depolarization in cultured hippocampal neurons results in the formation of new dendritic spines and filopodia (Wu et al., 2001). Increases in both MAPK activity and dendritic spine density have also been reported following NMDA receptor activation in hippocampal cultures (Goldin and Segal, 2003). Interestingly in this study, the ability of leptin to promote dendritic morphogenesis was dependent on the activation of putative synaptic NR2A-, but not extrasynaptic NR2B-, containing NMDA receptors. Our previous studies have shown that leptin facilitates hippocampal NMDA receptor function via a combination of PI 3-kinase and MAPK activation (Shanley et al., 2001). In contrast, leptin facilitation of NR2B-NMDA receptor-mediated responses in cerebellar granule cells requires the activation of a MAPK-dependent signaling cascade (Irving et al., 2006). This suggests not only that divergent signaling pathways couple leptin receptors to NMDA receptors with distinct molecular composition, but also that this coupling mechanism varies between different brain regions. Thus, it is possible that activation of MAPK by leptin results in selective facilitation of hippocampal NR2A-containing NMDA receptors that in turn promotes increases in dendritic filopodia.

There is mounting evidence that the hormone leptin plays a key modulatory role in hippocampal synaptic plasticity (Shanley et al., 2001; Li et al., 2002; Wayner et al., 2004; Harvey et al., 2006). Moreover, several lines of evidence suggest that structural remodeling of dendrites and/or synapses is a potential mechanism underlying hippocampal LTP (Yuste and Bonhoeffer, 2001; Toni et al., 1999). In hippocampal slices, exposure to leptin per se does not increase the efficacy of synaptic transmission, but it does facilitate NMDA receptor-dependent LTP (Shanley et al., 2001). In cultured hippocampal neurons the ability of leptin to promote morphogenesis is also dependent on NMDA receptor activation. Cultured neurons are generally more excitable than acute slices and display a higher frequency of mEPSCs. Thus, basal NMDA receptor activity is likely to be sufficient to support leptin-dependent synaptic plasticity, without the requirement for evoked synaptic transmission.

Leptin is known to circulate in the plasma in amounts proportional to body weight and it is capable of being transported to all regions of the brain via saturable transport across the blood–brain barrier (Banks et al., 1996). Moreover, the rate of leptin transport varies between brain regions and is also dependent on the plasma levels of leptin. The maximal rate of leptin transport into the hippocampus occurs when serum leptin levels are higher (Banks et al., 2000). As leptin levels increase 5- to 10-fold during the first two postnatal weeks (Ahima et al., 1998), transport of leptin into the hippocampus is likely to be particularly high early in neuronal development. Moreover, in addition to crossing the blood–brain barrier, there is also evidence that leptin mRNA is expressed in a number of brain regions, including the hippocampus (Morash et al., 1999), suggesting that the actual concentration of leptin reaching hippocampal synapses may be derived from locally released leptin as well as leptin within the cerebrospinal fluid. Thus, it is likely that the concentrations of leptin used in this study correlate well with those reaching the hippocampus at this stage of neuronal development. Moreover, during the induction phase of LTP, it is likely that leptin released from adipocytes enters the brain and acts in concert with locally derived leptin to increase the likelihood of LTP at CA1 synapses by promoting rapid changes in hippocampal dendritic morphology.

Cognitive deficits are thought to be associated with leptin-insensitivity in rodents (Li et al., 2002) and are also prevalent in humans with obesity-related diseases such as type II diabetes (Gispen and Biessels, 2000). Recent studies have also linked alterations in the circulating levels of leptin to Alzheimer’s disease, where patients with this disorder have lower than normal levels (Power et al., 2001). In addition, leptin attenuates the plasma levels of amyloid β in transgenic mice that over-express amyloid β. Thus, as obesity and related diseases are thought to result from resistance to leptin (Banks, 2004), it is likely that this also contributes to the cognitive deficits found in diabetics and may play a role in the impaired cognitive function associated with neurodegenerative disorders. The ability of leptin to promote rapid changes in neuronal morphology may be critical for its role in both normal CNS function and in neurological diseases associated with leptin resistance and cognitive deficits.

## Experimental methods

### Hippocampal cell culture and transfection

Cultures of hippocampal neurons were prepared as described previously (Shanley et al., 2001; 2002). Briefly, neonatal Sprague Dawley rats (1–3 days old) were killed by cervical dislocation in accordance with Schedule 1 of the United Kingdom Government Animals (Scientific Procedures) Act, 1986. The hippocampi were removed and following washing in standard HEPES-buffered saline comprising of (in mM) NaCl 135; KCl 5; CaCl_2_ 1; MgCl_2_ 1; HEPES 10; and d-glucose 25 at pH 7.4 were treated with a mixture of protease type X and type XIV (both at 0.5 mg/ml; Sigma) for 25 min at room temperature. Dissociated cells were plated onto sterile culture dishes (Falcon 3001), pretreated with poly-l-lysine (20 μg ml^− 1^ for 1–2 h). Cultures were maintained in serum replacement medium (SR2; Sigma) in a humidified atmosphere of 5% CO_2_ and 95% O_2_ at 37 °C for up to 3 weeks. Treatment with leptin was carried out in HEPES-buffered saline at room temperature (for exposures up to 30 min) or in culture media at 37 °C (for longer duration exposure times).

Hippocampal neurons were transiently transfected with GFP using the cationic lipid, Lipofectamine 2000 (Invitrogen Corporation, Carlsbad, CA, USA). Briefly, neurons up to 12 DIC were placed in 1.5 ml of conditioned medium for 30 min at 37 °C. The Lipofectamine/DNA complex was formed immediately by the addition of 2 μl of GFP-actin DNA (1 μg/μl) and 2 μl of Lipofectamine 2000 to 100 μl of Opti-MEM 1 (Invitrogen Corporation). Neurons were then incubated with this complex for 1 h in a humidified atmosphere of 5% CO_2_ and 95% O_2_. Following incubation, neurons were washed twice with MEM and then returned to normal conditioned medium. Expression of the EGFP construct was optimal 1–2 days after transfection, and a transfection rate of around 20% was observed. In transfected neurons, dendrites and axons as well as filopodia were clearly labeled.

For leptin receptor siRNA studies, hippocampal cells (6–7 DIC) were transfected with either EGFP cDNA (1 μg/μl) + prK5 (empty control vector; 1 μg/μl) or EGFP cDNA (1 μg/μl) + leptin receptor siRNA (Dharmacon; 1 μg/μl) according to the manufacturer’s instructions and were used 1–2 days after transfection. Dharmacon ON-TARGET*plus SMART*pool siRNA reagent (Thermo Fisher Scientific, Lafayette, CO) targeting LEPR (NM_012596) was used in these studies. The levels of leptin receptor expression in transfected neurons was assessed by comparing leptin receptor immunolabeling (see methods below) in transfected (GFP-positive) neurons relative to non-transfected (GFP-negative) neurons.

### Visualization of actin filaments

In order to examine the morphology of the actin-rich structures in fixed hippocampal neurons, Alexa 488-conjugated phalloidin, a specific marker that binds to polymerized actin filaments, was used. In these studies neurons were fixed (with 4% paraformaldehyde; 10 min), permeabilized (with 0.1% Triton X-100) and blocked with 10% blocking milk for 15 min. Cells were then incubated with Alexa 488-conjugated phalloidin (1:250 dilution) for 30 min at room temperature. Neurons were then washed twice in HEPES-buffered saline and actin staining was visualized using a Zeiss LSM510 confocal microscope (excitation 488 nm; emission 505–550 nm). The intensity of staining was determined off-line using Lasersharp software (Carl Zeiss). Analysis lines (50 μm) were drawn along randomly selected somatic and dendritic regions and the mean fluorescence intensity was calculated. Data were obtained from at least 3 cells selected at random for each condition. Within a given experimental series all conditions for capturing images, including illumination intensity and photomultiplier gains, were constant. In order to allow for quantification of experimental data obtained from separate days, the data were normalized relative to the mean fluorescence intensity measured in the control neurons for each day.

### Immunocytochemistry

Before labeling, hippocampal cultures were washed in HBS, fixed with paraformaldehyde (4%; 10 min) and permeabilized with Triton X-100 (0.1%; 5 min). To determine the distribution of ObR, neurons were incubated with an anti-goat polyclonal antibody (1:100 dilution) directed against the C-terminal domain of ObR (Santa Cruz Biotechnology, Santa Cruz, CA, USA) for 60 min as described previously (Shanley et al., 2002). In order to visualize staining, neurons were incubated with an Alexa 488-conjugated donkey anti-goat secondary antibody (1:250 dilution; Jackson ImmunoResearch, West Grove, PA, USA) for 30 min. For dual labeling studies, neurons were also incubated with monoclonal antibodies directed against synapsin-1 (BD Biosciences, Oxford, UK) or either NR2A (1:100 dilution) or NR2B (1:100 dilution; both from Invitrogen Corporation) for 1 h, followed by treatment with a donkey anti-mouse Cy3-conjugated secondary antibody (1:250 dilution; Molecular Probes, Eugene, OR, USA) for a further 30 min, respectively. All antibodies were prepared in HBS and experiments were performed at room temperature. In the absence of primary antibody, no labeling was observed following incubation with any of the secondary antibodies. In control experiments, leptin receptor immunoreactivity was blocked by prior incubation of primary antibody with control peptide (200 μg ml^− 1^). A Zeiss LSM510 laser scanning confocal microscope was used to visualize and capture images. Laser lines of 488 nm and 543 nm were used to excite Alexa 488 and Cy3, respectively. Dual labeling images were obtained in multi-tracking mode using a 15-s scan speed. All immunocytochemical studies were performed on at least two sets of cultures prepared from different rats.

### Real-time confocal imaging and analysis

Time-lapse confocal imaging of individual filopodium from live GFP-transfected neurons was performed using a Zeiss LSM510 meta laser scanning confocal microscope (Carl Zeiss Ltd, Rugby, UK) using a × 40 magnification water immersion Plan-Apochromat objective and sequential line scanning mode. Neuronal cultures were maintained in a modified Mini-Chamber (Luigs & Neumann, Ratingen Germany) at room temperature. In time series mode, full Z-stack images were acquired at 3 min intervals using minimal 488 nm excitation (1.9-s scan speed at 512 × 512 pixels, 1–3% maximal argon laser power) for the duration of recordings (30 min) in control conditions (in normal HEPES-buffered saline) and subsequently for a further 30 min following addition of leptin (50 nM). The Z-stack function (1.5–2.5 μm sections; iris aperture of 3–5 μm) was used in conjunction with the time series mode to enable 3D reconstruction of live neurons. Proximal dendrites, identified as MAP2-positive processes extending from the cell body, were selected for analysis of the length and number of dendritic filopodia. The length of an individual filopodium was defined as the linear distance between its tip and its base and the lengths of individual filopodia were determined manually using Zeiss Lasersharp software. To measure the motility of dendritic filopodia with time, the length of randomly selected filopodia was measured in each frame and the relative change in filopodia length (compared to the filopodia length at time point zero) was calculated for the duration of experiments as the total change in filopodia length divided by the time (μm/min; Ziv and Smith, 1996). Only time points in which filopodial length was greater than 0.6 μm in one or both of two consecutive images were included in the analyses, whereas time points where the length of filopodia was less than 0.6 μm in two consecutive images were excluded.

### Materials

Recombinant human leptin (Sigma, St. Louis, MO, USA) prepared in 0.01–0.02% bovine serum albumin as a carrier was used in all experiments. LY294002, wortmannin, U0126 (Calbiochem, La Jolla, USA) and D-AP5, ifenprodil, tetrodotoxin, PD98059 (Tocris Cookson, Baldwin, MO, USA) were all obtained commercially.

### Statistical analyses

All data are expressed as means ± SEM and statistical analyses were performed using paired *t*-test (two-tailed; 95% confidence interval) for comparison of means or two-way ANOVA (analysis of variance) with Tukey’s post hoc test for comparisons between multiple groups (unless otherwise stated). *P* < 0.05 was considered significant.

## References

[bib32] Ahima R.S., Prabakaran D., Flier J.S. (1998). Postnatal leptin surge and regulation of circadian rhythm of leptin by feeding. Implications for energy homeostasis and neuroendocrine function. J. Clin. Invest..

[bib1] Ahima R.S., Bjorbaek C., Osei S., Flier, J.S., Regulation of neuronal and glial proteins by leptin: implications for brain development. J.S. (1999). Endocrinology.

[bib3] Antonova I., Arancio O., Trillat A.C., Wang H.G., Zablow L., Udo H., Kandel E.R., Hawkins R.D. (2001). Rapid increase in clusters of presynaptic proteins at onset of long-lasting potentiation. Science.

[bib2] Banks W.A, (2004). The many lives of leptin. Peptides.

[bib5] Banks W.A., Kastin A.J., Huang W., Jaspan J.B., Maness L.M. (1996). Leptin enters the brain by a saturable system independent of insulin. Peptides.

[bib4] Banks W.A., Clever C.M., Farrell C.L. (2000). Partial saturation and regional variation in the blood-to-brain transport of leptin in normal weight mice. Am. J. Physiol: Endocrinol. Metab..

[bib6] Barria A., Malinow R. (2002). Subunit-specific NMDA receptor trafficking to synapses. Neuron.

[bib11] Bates S.H., Myers M.G. (2004). The role of leptin receptor signaling in feeding and neuroendocrine function. Trends Endocrinal. Metab..

[bib7] Bjorbaek C., Buchholz R.M., Davis S.M., Bates S.H., Pierroz D.D., Gu H., Neel B.G., Myers M.G., Flier J.S. (2001). Divergent roles of SHP-2 in ERK activation by leptin receptors. J. Biol. Chem..

[bib8] Blenis J. (1993). Signal transduction via the MAP kinases: proceed at your own RSK. Proc. Natl. Acad. Sci. U. S. A..

[bib9] Bouret S.G., Draper S.J., Simerly R.B. (2004). Trophic action of leptin on hypothalamic neurons that regulate feeding. Science.

[bib10] Chun D., Gall C.M., Bi X., Lynch G. (2001). Evidence that integrins contribute to multiple stages in the consolidation of long-term potentiation in rat hippocampus. Neuroscience.

[bib12] Collingridge G.L., Isaac J.T., Wang Y.T. (2004). Receptor trafficking and synaptic plasticity. Nat. Rev., Neurosci..

[bib13] Dillon C., Goda Y. (2005). The actin cytoskeleton: integrating form and function at the synapse. Annu. Rev. Neurosci..

[bib14] Elmquist J.K., Bjorbaek C., Ahima R.S., Flier J.S., Saper C.B. (1998). Distributions of leptin receptor mRNA isoforms in the rat brain. J. Comp. Neurol..

[bib15] Elmquist J.K., Maratos-Flier E., Saper C.B., Flier J.S. (1998). Unravelling the central nervous system pathways underlying responses to leptin. Nat. Neurosci..

[bib45] Ethell I.M., Pasquale E.B. (2005). Molecular mechanisms of dendritic spine development and remodeling. Prog. Neurobiol..

[bib16] Fiala J.C., Feinberg M., Popov V., Harris K.M. (1998). Synaptogenesis via dendritic filopodia in developing hippocampal area CA1. J. Neurosci..

[bib17] Fischer M., Kaech S., Knutti D., Matus A. (1998). Rapid actin-based plasticity in dendritic spines. Neuron.

[bib18] Fischer M., Kaech S., Wagner U., Brinkhaus H., Matus A. (2000). Glutamate receptors regulate actin-based plasticity in dendritic spines. Nat. Neurosci..

[bib19] Freidman J.M., Halaas J.L. (1998). Leptin and the regulation of body weight in mammals. Nature.

[bib20] Fujioka Y., Matozaki T., Noguchi T., Iwamatsu A., Yamao T., Takahashi N., Tsuda M., Takada T., Kasuga M. (1996). A novel membrane glycoprotein, SHPS-1, that binds the SH2-domain-containing protein tyrosine phosphatase SHP-2 in response to mitogens and cell adhesion. Mol. Cell. Biol..

[bib21] Fukazawa Y., Saitoh Y., Ozawa F., Ohta Y., Mizuno K., Inokuchi K. (2003). Hippocampal LTP is accompanied by enhanced F-actin content within the dendritic spine that is essential for late LTP maintenance in vivo. Neuron.

[bib22] Gispen W.H., Biessels G.J. (2000). Cognition and synaptic plasticity in diabetes mellitus. Trends Neurosci..

[bib23] Goldin M., Segal M. (2003). Protein kinase C and ERK involvement in dendritic spine plasticity in cultured rodent hippocampal neurons. Eur. J. Neurosci..

[bib24] Hakansson M.-L., Brown H., Ghilardi N., Skoda R.C., Meister B. (1998). Leptin receptor immunoreactivity in chemically-defined target neurons of the hypothalamus. J. Neurosci..

[bib25] Halpain S., Hipolito A., Saffer L. (1998). Regulation of F-actin stability in dendritic spines by glutamate receptors and calcineurin. J. Neurosci..

[bib26] Harvey J. (2003). Leptin: a multifaceted hormone in the central nervous system. Mol. Neurobiol..

[bib27] Harvey J., Shanley L.J., O’Malley D., Irving A. (2005). Leptin: a potential cognitive enhancer?. Biochem. Soc. Trans..

[bib28] Harvey J., Solovyova N., Irving A. (2006). Leptin and its role in hippocampal synaptic plasticity. Prog. Lipid Res..

[bib29] Irving A.J., Wallace L., Durakoglugil D., Harvey J. (2006). Leptin enhances NR2B-mediated *N*-methyl-d-aspartate responses via a mitogen-activated protein kinase-dependent process in cerebellar granule cells. Neuroscience.

[bib30] Jacob R.J., Dziura J., Medwick M.B., Leone P., Caprio S., During M., Shulman G.I., Sherwin R.S. (1997). The effect of leptin is enhanced by microinjection into the ventromedial hypothalamus. Diabetes.

[bib31] Karsenty G. (2001). Leptin controls bone formation through a hypothalamic relay. Rec. Prog. Horm. Res..

[bib33] Li X.L., Aou S., Oomura Y., Hori N., Fukunaga K., Hori T. (2002). Impairment of long-term potentiation in leptin receptor deficient rodents. Neuroscience.

[bib34] Liu L., Wong T.P., Pozza M.F., Lingenhoehl K., Wang Y., Sheng M., Auberson Y.P., Wang Y.T. (2004). Role of NMDA receptor subtypes in governing the direction of hippocampal synaptic plasticity. Science.

[bib35] Maletic-Savatic M., Malinow R., Svoboda K. (1999). Rapid dendritic morphogenesis in CA1 hippocampal dendrites induced by synaptic activity. Science.

[bib36] Massey P.V., Johnson B.E., Moult P.R., Auberson Y.P., Brown M.W., Molnar E., Collingridge G.L., Bashir Z.I. (2004). Differential roles of NR2A and NR2B-containing NMDA receptors in cortical long-term potentiation and long-term depression. J. Neurosci..

[bib37] Matus A. (2000). Actin-based plasticity in dendritic spines. Science.

[bib65] McAllister A.K., Lo D.C., Katz L.C. (1995). Neurotrophins regulate dendritic growth in developing visual cortex. Neuron.

[bib38] McKinney R.A., Capogna M., Durr R., Gahwiler B.H., Thompson S.M. (1999). Miniature synaptic events maintain dendritic spines via AMPA receptor activation. Nat. Neurosci..

[bib39] Morash B., Li A., Murphy P.R., Wilkinson M., Ur E. (1999). Leptin gene expression in the brain and pituitary gland. Endocrinology.

[bib40] Morash B., Wilkinson D., Murphy P., Ur E., Wilkinson M. (2001). Developmental regulation of leptin gene expression in rat brain and pituitary. Mol. Cell. Endocrinol..

[bib41] Munno D.W., Syed N.I. (2003). Synaptogenesis in the CNS: an odyssey from wiring together to firing together. J. Physiol..

[bib42] Niblock M.M., Brunso-Bechtold J.K., Riddle D.R. (2000). Insulin-like growth factor 1 stimulates dendritic growth in primary somatosensory cortex. J. Neurosci..

[bib43] Niswender K.D., Morton G.J., Stearns W.H., Rhodes C.J., Myers M.G., Schwartz M.W. (2001). Intracellular signalling. Key enzyme in leptin-induced anorexia. Nature.

[bib66] O'Malley D., Irving A.J., Harvey J. (2005). Leptin-induced dynamic alterations in the actin cytoskeleton mediate the activation and synaptic clustering of BK channels. FASEB.

[bib44] Pinto S., Roseberry A.G., Liu H., Diano S., Shanabrough M., Cai X., Friedman J.M., Horvath T.L. (2004). Rapid rewiring of arcuate nucleus feeding circuits by leptin. Science.

[bib46] Power D.A., Noel J., Collins R., O’Neill D. (2001). Circulating leptin levels and weight loss in Alzheimer’s disease patients. Dement. Geriatr. Cogn. Disord..

[bib47] Schinder A.F., Poo M. (2000). The neurotrophin hypothesis for synaptic plasticity. Trends Neurosci..

[bib48] Shanley L.J., Irving A.J., Harvey J. (2001). Leptin enhances NMDA receptor function and modulates hippocampal synaptic plasticity. J. Neurosci..

[bib49] Shanley L.J., O’Malley D., Irving A.J., Ashford M.L., Harvey J. (2002). Leptin inhibits epileptiform-like activity in rat hippocampal neurones via PI 3-kinase-driven activation of BK channels. J. Physiol..

[bib50] Shi Y., Ethell I.M. (2006). Integrins control dendritic spine plasticity in hippocampal neurons through NMDA receptor and Ca^2+^/calmodulin-dependent protein kinase II-mediated actin reorganization. J. Neurosci..

[bib51] Smart F.M., Halpain S. (2000). Regulation of dendritic spine stability. Hippocampus.

[bib52] Tartaglia L.A., Dembski M., Weng X., Deng N., Culpepper J., Devos R., Richards G.J., Campfield L.A., Clark F.T., Deeds J. (1995). Identification and expression cloning of a leptin receptor, OB-R. Cell.

[bib53] Tashiro A., Dunaevsky A., Blazeski R., Mason C.A., Yuste R. (2003). Bidirectional regulation of hippocampal mossy fiber filopodial motility by kainate receptors: a two-step model of synaptogenesis. Neuron.

[bib59] Thomas G.M., Huganir R.L. (2004). MAPK cascade signalling and synaptic plasticity. Nat. Rev. Neurosci..

[bib54] Toni N., Buchs P.A., Nikonenko I., Bron C.R., Muller D. (1999). LTP promotes formation of multiple spine synapses between a single axon terminal and a dendrite. Nature.

[bib55] Tovar K.R., Westbrook G.L. (1999). The incorporation of NMDA receptors with a distinct subunit composition at nascent hippocampal synapses in vitro. J. Neurosci..

[bib57] Tyler W.J., Pozzo-Miller L. (2003). Miniature synaptic transmission and BDNF modulate dendritic spine growth and form in rat CA1 neurones. J. Physiol..

[bib56] Tyler W.J., Alonso M., Bramham C.R., Pozzo-Miller L.D. (2002). From acquisition to consolidation: on the role of brain-derived neurotrophic factor signaling in hippocampal-dependent learning. Learn. Mem..

[bib58] Valerio A., Ghisi V., Dossena M., Tonello C., Giordano A., Frontini A., Ferrario M., Pizzi M., Spano P., Carruba M.O., Nisoli E. (2006). Leptin increases axonal growth cone size in developing mouse cortical neurons by convergent signals inactivating glycogen synthase kinase-3beta. J. Biol. Chem..

[bib60] Wayner M.J., Armstrong D.L., Phelix C.F., Oomura Y. (2004). Orexin-A (Hypocretin-1) and leptin enhance LTP in the dentate gyrus of rats in vivo. Peptides.

[bib61] Williams K. (1993). Ifenprodil discriminates subtypes of the *N*-methyl-d-aspartate receptor: selectivity and mechanisms at recombinant heteromeric receptors. Mol. Pharmacol..

[bib62] Wu G.Y., Deisseroth K., Tsein R.W. (2001). Spaced stimuli stabilize MAPK pathway activation and its effects on dendritic morphology. Nat. Neurosci..

[bib63] Yuste R., Bonhoeffer T. (2001). Morphological changes in dendritic spines associated with long-term synaptic plasticity. Annu. Rev. Neurosci..

[bib64] Ziv N.E., Smith S.J. (1996). Evidence for a role of dendritic filopodia in synaptogenesis and spine formation. Neuron.

